# Remembering Mary (1917 to 2008): editorial introduction to the thematic series on the life and lifework of Mary Mandels, first lady of cellulase research

**DOI:** 10.1186/1754-6834-2-23

**Published:** 2009-09-01

**Authors:** Edward A Bayer

**Affiliations:** 1 Department of Biological Chemistry, The Weizmann Institute of Science, Rehovot, Israel

## Abstract

Editorial introduction to the thematic series on the life and lifework of Mary Mandels.

## Commentary

On 17 February 2008, Mary Mandels passed away, and so ended an era.

As I remember Mary, my thoughts race back to the summer of 1994 when I was invited to attend a workshop on the nomenclature of cellulases and other related enzymes, organised by Sharon Shoemaker, which took place in the idyllic setting of Fallen Leaf Lake (Lake Tahoe Basin, CA, USA). Although I had been active in the field for over a decade, this workshop was my first face-to-face meeting with many of my colleagues in the cellulase field, most of whom were destined to become my dear friends. Mary was there, even then long retired, a link to the past...

My marathon travel experience from Tel Aviv to Fallen Leaf Lake included a transatlantic flight to JFK airport, New York, followed by a transcontinental flight to San Francisco International Airport. There, I found myself in the United Express annex, a couple of hours before my flight to Sacramento, where hoards of travellers awaited their flights to various obscure cities and towns in California. In true *Théâtre de l'Absurde *style, flights were announced, overbooked seats negotiated, hustle and bustle, a sea of commotion. I stood on the side, awestruck by the follies and foibles of the human comedy before me. When our flight was announced, we were led by a flag-carrying agent out to the small aircraft in single file and found our seats. I knew we were in trouble when the pilot appeared, scanned the group of passengers, and started pointing at the heftier ones, saying, 'You sit here, you sit over there...' in order to balance out the plane. After a particularly bumpy and uncomfortable ride through the clouds, I found myself in Sacramento. I took a cab to my hotel in Davis, arriving in the late morning hours, knowing I had to find a way to stay awake all day to counter jet lag. I met David Wilson and Tuula Teeri at the hotel and introduced myself to them. Later in the day, I went to the UC Davis campus, and found Roy Doi's office; my first of many happy encounters with Roy. In the evening, I again saw Tuula in the hotel and asked her if she'd like to go to the movies. Somehow, I managed to keep awake until midnight.

The next day I found myself among 40 or so strangers, but potential future friends, on the bus towards the Lake Tahoe area. We arrived a couple of hours later at the Stanford Sierra Camp, the site of the workshop. I was assigned to room with Doug Eveleigh, and even if you'd try for years, you could never succeed in finding a friendlier, wittier, more dynamic or colourful roommate! And the meeting itself was laden with friendly, witty, dynamic and colourful personalities.

As I walked among the weathered wooden buildings overlooking the beautifully picturesque Fallen Leaf Lake, I was suddenly accosted by Mike Sinnott, who looked quite lost with various types of garbage in his hands. He asked me, 'Do you know where I can find the 'politically correct' trash cans?'

I knew then that I was going to fit in and that I was in for an interesting meeting!

Other participants at the workshop included Mike Himmel, Bernard Henrissat, Martin Schulein, Jean-Pierre Belaich, Pedro Alzari, Frederic Barras, Jennifer Thomson, Marc Claeyssens, Kunio Ohmiya, Mike Penner and Tony Warren. The discussions focused on how to organise and classify the diverse types of cellulases in nature. The major position of the participants maintained that the cellulases and related enzymes fail to conform to the normal classification schemes, designated by their Enzyme Commission (EC) number as recommended by the International Union of Biochemistry and Molecular Biology (IUBMB), and that a different approach should be enforced. For the cellulases and related enzymes (the glycoside hydrolases) collectively comprise unique enzyme systems that decompose a unique type of substrate: the intricate conglomerate of plant cell wall polysaccharides. The single major outcome of the meeting was a publication describing a new scheme for classifying the glycoside hydrolases [1], which became a forerunner of the establishment of the CAZy (for 'Carbohydrate-Active enZymes') database [2] and website [3].

During a break, we were led down to the lake, where kayaks were available. This was my first experience with kayaking and I took to it naturally, kayaking all over the place with ease. I saw Mike Himmel on the dock watching me. I yelled to him, 'Mike, this is great!' Mike gingerly slipped into a kayak that immediately overturned. Soaked and embarrassed, Mike climbed out of the water back onto the dock to dry out his wallet and its contents. My first impulse was to laugh, and I did so brazenly and without inhibition, while continuing to display my newly found kayaking skills. I don't think I was even conscious of Mike's icy glare. Fortunately, and despite my unforgivable indiscretions, Mike and I have since become true scientific and personal friends, and our friendship has only strengthened greatly through the years.

**Figure 1:**
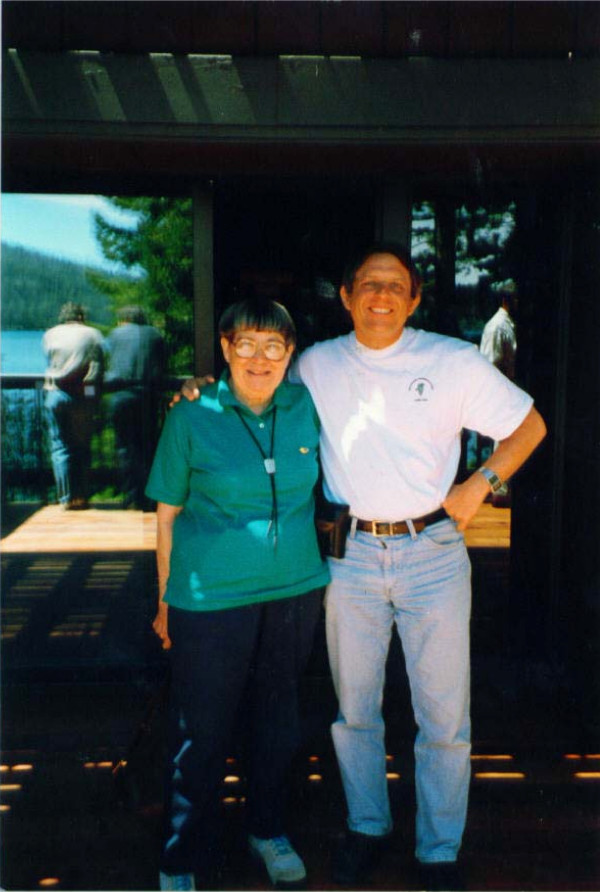
**Mary and me**. Mary Mandels (left) and the author at Fallen Leaf Lake, June 1994.

Mary was a calming and modest presence at the meeting. Everyone was conscious of her place in the history of the cellulases. I remember her soft smile. I was fortunate to have my picture taken together with her (Figure 1).

Mary's name is indelibly connected to Elwyn T Reese, who isolated the causative agent responsible for the 'jungle rot' that plagued the cotton tents of the allied forces in the South Pacific during World War II. The fungus was identified as *Trichoderma *'*viride*' strain QM 6a, which was later renamed *Trichoderma reesei *QM 6a, in honour of its discoverer. As fortune would have it, though, the isolate eventually turned out to be a known and separate fungal species (and even genus), and has now been reclassified as *Hypocrea jecorina *[4], a fungus first described well over a century ago [5]. Honour and recognition are but fleeting gifts. Elwyn never lived to see this turn of fate, but Mary did.

Mary's association with Elwyn Reese spanned over a quarter of a century. About half her publications and a quarter of his are common. Their work established the initial types of cellulase assays from scratch, as well as unique approaches to the investigation of microbial degradation of model cellulosic substrates. Many of these assays and approaches are still in use today, half a century later. Mary Mandels and Elwyn T Reese were true pioneers.

Today, of course, the renewed 'advent' of the biomass to biofuels initiatives worldwide (and the accompanying funding opportunities) has spawned renewed interest in the cellulases and the microbes which produce them [6-8]. We have witnessed ups and downs in the past several decades; the popularity of the field seems to have been directly related to dramatic events in the Middle East, the price of petroleum and the whims and agendas of foreign powers. For one reason or another, this area has been decidedly underfunded over the years and we have lost many of the groups that have had exquisite expertise in the area of cellulases and their parent micro-organisms. Some scientists have left the area due to lack of funds and the attraction to other 'more popular and profitable' fields, in which the funding potential has been much more productive. Others have succumbed to the effects of time and have retired from science altogether. Nevertheless, still others have remained, captivated by the remarkable qualities of the enzymes and the uniqueness of the bacterial and fungal systems that produce them. In general, the remaining groups have survived by eking out their finances and struggling to keep their labs afloat.

The newfound funding opportunities have now attracted both young and more established scientists to the field, who are, by definition, novices. New blood is good for any field, and indeed the funding bodies have responded in kind, with a strong emphasis on new ideas. However, the singularity of these enzymes, the microbes and their ecosystems reign, and to ignore the accumulated knowledge and experience of those scientists who have stubbornly continued in the field over the past several decades would be imprudent, wasteful and counterproductive. Newcomers to the field are constantly misinterpreting their data or rediscovering previously established findings that have been published decades ago, sometimes in obscure places inaccessible to contemporary electronic resources. Novel ideas should be tempered by knowledge and experience.

Remembering Mary is to connect ourselves to the onset of the modern era of cellulase research. The thematic series on the life and lifework of Mary Mandels begins with an authoritative historical obituary, which includes vintage pictures of Mary throughout her years at the US Army cellulase laboratory at Natick. This is followed by one of Mary's benchmark publications, and a previously unpublished commentary on Mary's views of the US military establishment after nearly five decades of experience (written over two decades ago). This is Mary talking to us posthumously: again, a voice from the past. Listen to what she has to say! Sound familiar??!! This is not necessarily a commentary on the military establishment *per se*, but a treatise on our society and human nature in general. One of Mary's seminal articles [9] is also included in this series as a republication, in order to remind newcomers to the field how initial research on cellulase-mediated degradation of cellulosic substrates was converted in a creative way into a science. Other contributions will be limited to authors who have worked on the cellulase system of *Trichoderma reesei *turned *Hypocrea jecorina*, many of whom had worked with Mary at one point in their careers, and thus serve as additional direct links to the past. The first of these is an article by Christian Kubicek and colleagues, which summarises the current status of regulation of cellulase biosynthesis in this fungus and outlines strategies for improving cellulase production. The series will be published sporadically over a period of the next several months, the overarching theme of which remains the connection with Mary, her life's work, and the emergence of modern day cellulase research.
